# MicroRNA regulation of natural killer cells

**DOI:** 10.3389/fimmu.2013.00044

**Published:** 2013-02-28

**Authors:** Ryan P. Sullivan, Jeffrey W. Leong, Todd A. Fehniger

**Affiliations:** Division of Oncology, Department of Medicine, Washington University School of MedicineSt. Louis, MO, USA

**Keywords:** microRNA, miRNA, natural killer

## Abstract

Natural killer (NK) cells are innate immune lymphocytes critical for host defense against viral infection and surveillance against malignant transformation. MicroRNAs (miRNAs) are a family of small, non-coding RNAs that regulate a wide variety of cellular processes. Recent advances have highlighted the importance of miRNA-mediated post-transcriptional regulation in NK cell development, maturation, and function. This review focuses on several facets of this regulatory mechanism in NK cells: (1) the expressed NK cell miRNA transcriptome; (2) the impact of total miRNA deficiency on NK cells; (3) the role of specific miRNAs regulating NK cell development, survival, and maturation; (4) the intrinsic role of miRNAs regulating NK cell function, including cytokine production, proliferation, and cytotoxicity; and (5) the role of NK cell miRNAs in disease. Currently our knowledge of how miRNAs regulate NK cell biology is limited, and thus we also explore key open questions in the field, as well as approaches and techniques to ascertain the role of individual miRNAs as important molecular regulators.

## INTRODUCTION

Natural killer (NK) cells are innate immune lymphocytes that develop from the common lymphoid progenitor in the bone marrow; however, NK cells are defined by a unique biological program that is distinct from the adaptive T and B lymphocytes which develop from that same progenitor (Yokoyama et al., 2004). NK cell development proceeds through a series of intermediates that are dependent on growth factors and cytokines, distinct from B and T cell development, to support their development and homeostasis, especially IL-15 (Colucci et al., 2003; Freud and Caligiuri, 2006). Furthermore, NK cells reconstitute after hematopoietic stem cell transplantation more rapidly than other lymphocytes (Storek et al., 2008), further attesting to their uniqueness as a lymphoid lineage. However, although the distinctions between NK cells and other lymphocytes are well known, the molecular events and programs that define NK cells are incompletely understood, and remain an important area of NK cell investigation.

In mice, NK cells are identified as lymphocytes that express two activating receptors: NK1.1 (C57Bl/6) and NKp46 (virtually all mouse background strains), and lack CD3 and CD19 expression (Colucci et al., 2003). In humans, NK cells are distinguished by their expression of CD56, and a lack of CD3 and rearranged T cell receptors. NK cells begin their development in the bone marrow, where they are subject to an education process, in which the NK cells are licensed to full functionality by becoming tuned to the “normal” levels of cell surface ligands, primarily major histocompatibility complex (MHC) class I or related molecules (Joncker and Raulet, 2008; Jonsson and Yokoyama, 2009). Murine NK cells undergo further maturation in peripheral lymphoid organs, marked by expression of CD27, CD11b, and CD43 (Chiossone et al., 2009). Human NK cells in the periphery may further be divided into subsets by their expression of CD56 and CD16 (FCγRIIIa): the functionally and developmentally distinct CD56^bright^ and CD56^dim^ populations (Caligiuri, 2008).

Mature peripheral NK cells defend the host from pathogens and mediate anti-tumor responses (Yokoyama et al., 2004; Caligiuri, 2008; Di Santo, 2008; Lanier, 2008). One way that NK cells protect the host is by detecting abnormal surface receptor repertoires on target cells, through loss of self-defining proteins and/or gain of abnormal or induced self-proteins. Typical inhibitory NK cell receptor ligands include MHC class I molecules, which are often down-regulated on target tumor and infected cells, and “stress” ligands such as retinoic acid early inducible-I (Rae-I) and UL16 binding protein 1 (ULBP1), which can activate NK cells and are often up-regulated on these target cells. It is the relative levels of these inhibitory and activating signals that determine whether or not the NK cell is triggered to respond to a potential target cell (Lanier, 2005; Bryceson and Long, 2008). Once triggered, NK cells then mediate cytotoxicity against these cells through the release of cytotoxic granules, as well as produce various cytokines and chemokines that influence the developing immune response (Vivier et al., 2008). In addition to responding to ligands on target cells, NK cells express a number of cytokine receptors and can be activated or primed by pro-inflammatory cytokines for optimal effector function. Cytokine activation can also override inhibition mediated by some cell surface signals, and thus represents another important signal for NK cell functional capacity (Fehniger et al., 2007; Lee et al., 2007; Lucas et al., 2007; Caligiuri, 2008; Chaix et al., 2008).

Currently, our understanding of the basic molecular mechanisms regulating NK cell development, maturation, survival, and function is incomplete. A number of transcription factors have been identified that contribute to NK cell development and function (Ramirez and Kee, 2010; Hesslein and Lanier, 2011). Recently, studies have highlighted the role of post-transcriptional control, especially microRNAs (miRNAs), on the NK cell molecular program (Fehniger et al., 2007; Bezman et al., 2010; Sullivan et al., 2012). In this review, we highlight the current state of the field in miRNA-mediated control of NK cell development, survival, maturation, with a focus on global and specific miRNA deficiency and over-expression. As our knowledge of the molecular programs, including miRNAs, that regulate NK cells increases, this will lead to identification of novel molecules and pathways that may be manipulated to enhance or attenuate NK cell function.

## MicroRNAs

MicroRNAs are non-coding RNAs, many of which are highly conserved and regulate numerous cellular functions. miRNA repression is mediated primarily by targeting sites in the 3′UTR of mRNAs, leading to translational inhibition or causing mRNA degradation (He and Hannon, 2004). One report has indicated that some miRNAs can *increase *protein expression via binding to the 5′UTR (Vasudevan et al., 2007), but it remains unclear whether this is widely applicable.

**FIGURE 1:**
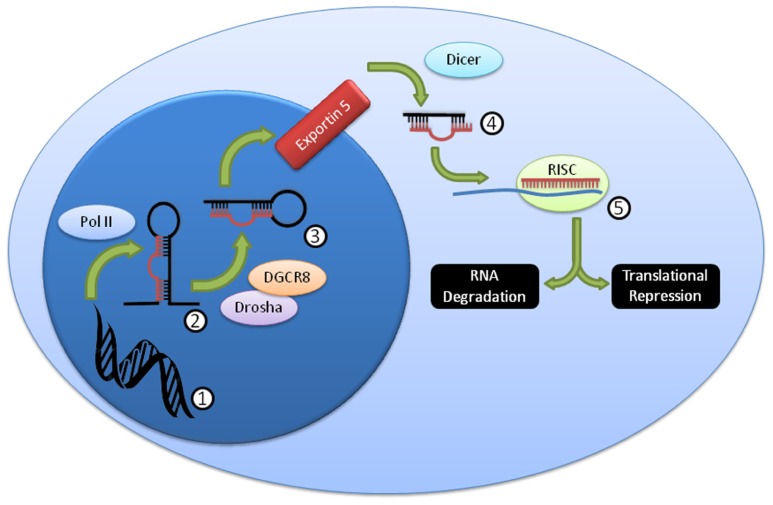
**Summary of canonical miRNA biogenesis and function**. DNA (1) is transcribed by PolII into the pri-miRNA (2), which is then further processed by Drosha/DGCR8 into the pre-miRNA (3). This pre-miRNA is exported from the nucleus through exportin 5, then processed by Dicer to the mature miRNA duplex (4). This duplex is unwound, and one strand, the mature miRNA, is loaded into the RISC complex (5), where it usually binds imperfectly to a target mRNA and mediates RNA degradation and/or translational repression.

Over 1,000 miRNA genes are predicted in humans and mice. miRNAs can be processed in a variety of ways (Winter et al., 2009), but the canonical pathway (**Figure 1**) consists of transcription from genomic sequence as long primary (pri-miRNA) transcripts that are “cropped” by the Drosha/Dgcr8 complex into precursor miRNAs (pre-miRNA) that have a characteristic stem-loop structure (Kim, 2005). The pre-miRNA is exported to the cytoplasm where it is “diced” by the Dicer complex (including Dicer1) into a mature 19–26 nucleotide miRNA. The mature miRNA is loaded into the RNA-induced silencing complex (RISC) that includes the Argonaute proteins, and thereby directs down-regulation of protein levels (Filipowicz et al., 2008; Liu, 2008; Wu and Belasco, 2008). While the exact mechanism of protein reduction remains controversial, it is likely that it occurs through both RNA degradation and translational repression pathways, perhaps with different miRNAs contributing to each pathway in different proportions. While perfect miRNA:mRNA complementarity (common in *C. elegans*) can lead to direct mRNA degradation, most miRNAs in higher organisms are imperfectly matched, and lead to mRNA degradation through deadenylation and decapping methods (Liu, 2008). Both processes are mediated by the “stem” of the “stem-loop,” the critical segment of the miRNA gene.

The role of miRNA-mediated regulation in antigen-specific T and B lymphocytes is well characterized. In these cells, development, survival, and function have been found to be controlled by miRNAs, data which are clearly supported by mouse models of miRNA gain- and loss-of-function (O’Connell et al., 2010b). While the data supporting critical regulation of NK cells by miRNAs are more exiguous (Di Santo, 2008), this review synthesizes recent studies that have provided the first insights into the expressed mouse and human NK cell miRNA transcriptome and mechanisms whereby miRNAs regulate NK cell development, homeostasis, and functional responses.

## THE NK CELL microRNA TRANSCRIPTOME

In contrast to T and B lymphocytes, there are few studies examining the miRNA profiles of human or mouse NK cells. The majority of studies that include NK cell expression data have focused on lymphocyte-wide comparisons, with an emphasis on identifying miRNAs with cell-specific functions (Landgraf et al., 2007; Rossi et al., 2011; Allantaz et al., 2012). As a result, many of these studies utilize miRNA microarrays or Sanger sequencing of small RNA clones. While these approaches provide the coverage necessary for identifying miRNA “signatures,” there is comparatively less miRNA detection depth and and/or dynamic range relative to unbiased next-generation small RNA sequencing. Additionally, next-generation sequencing provides novel miRNA identification, although at the tradeoff of higher cost per sample. Ideally, any accurate and reliable profiling data would utilize cross-platform validation of multiple biological samples.

**FIGURE 2:**
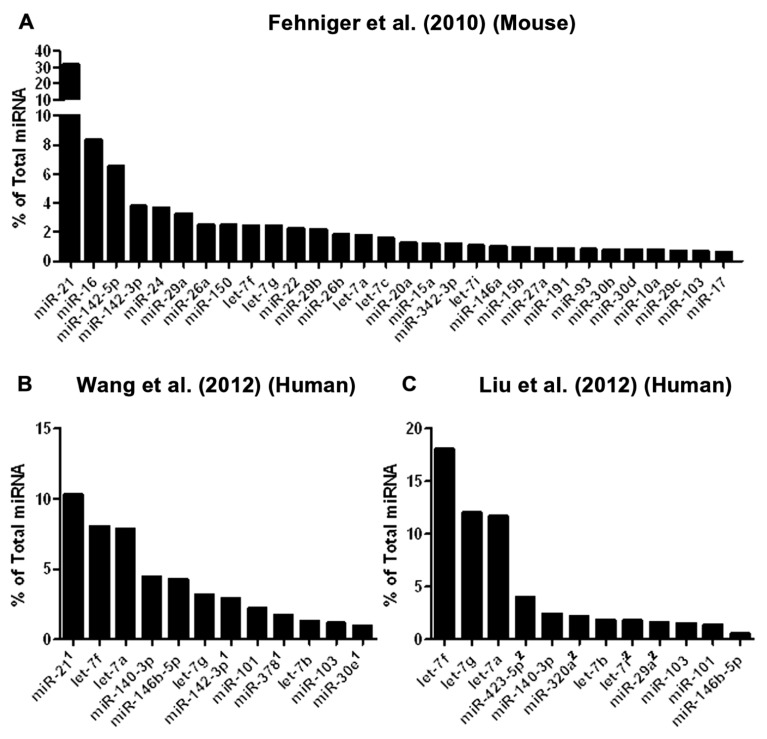
**Mouse and human NK cell miRNA expression**. **(A)** The top 30 miRNAs detected by Illumina sequencing in mouse splenic NK cells (Fehniger et al., 2010). **(B)** Top expressed miRNAs detected and enumerated by small RNA Illumina sequencing of resting human CD56^+^CD3^-^ in the Wang et al.’s 2012 study. **(C)** Top expressed miRNAs detected by small RNA Illumina sequencing in the Liu et al.’s 2012 study. The Wang et al.’s 2012 and Liu et al.’s 2012 data are limited to the miRNAs reported in the published paper, since the numerical data was not provided in supplemental files nor deposited in public databases. Liu et al.’s 2012 data were estimated from graphs included in the original paper. ^1^miRNA not reported in Liu et al. (2012). ^2^miRNA not reported in Wang et al. (2012).

To date, three studies have utilized next-generation sequencing to probe mouse or human NK cell miRNA expression (**Figure 2**). We identified the resting and 24-h IL-15-activated profile of murine splenic NK cells (Fehniger et al., 2010). This revealed that while >200 miRNAs were detectable in NK cells, the top 50 constituted >95% of the miRNA sequence content. The miRNA expression profile was supported by two independent next-generation sequencing platforms (Illumina and SOLiD), with validation by qPCR and microarrays. Overall, the expression of normalized miRNA read counts was similar between resting and 24-h IL-15-activated NK cells, with a small number of miRNAs significantly modulated, peaking around threefold. It will be important to extend these profiling studies to different types of mouse NK cells, including developmental and maturation intermediates, NK cells from distinct tissue compartments, and in the context of physiologic responses to infectious pathogens and tumor cells. A larger, more robust set of miRNA expression profiles will also facilitate comparison to other lymphocytes, and identify NK cell miRNA signatures which likely contribute to an NK cell-specific molecular program.

Two subsequent studies by Liu and colleagues and Wang and colleagues reported the miRNA profiles of resting and interferon-α (IFN-α; Wang et al., 2012), IL-2, IL-15, and IL-21 (Liu et al., 2012) activated bulk CD56^+^CD3^-^ human NK cells from peripheral blood. The resting human NK cell data sets show substantial variation from each other, thus indicating that human diversity may also be an important consideration during expression studies. Indeed, both groups that have reported human NK expression include data from only one resting human sample, which limits conclusions that can be drawn from comparing these particular data sets to each other. Additionally, the profiling from Wang et al. (2012) is challenging to compare to the other studies, since only the top 12 miRNAs were reported outside of their private activation-based analysis. Following activation, human NK cells appear to modestly up-regulate nearly half of all expressed miRNAs in response to IL-15, although these data rely on comparisons of resting and activated cells from separate donors (**Table 1**). The miRNAs that changed with activation demonstrated minimal overlap, suggesting activation-specific modulation or donor variation. Interestingly, NK cells activated with IFN-α respond by down-regulating a number of miRNAs, suggesting that miRNA changes are dependent on the mode of stimulation. Such global changes in miRNA may also reflect time point of measurement or normalization biases, since the absolute miRNA content of a cell can remain unchanged even as other small RNA content increases. Indeed, 12-h IFN-α-activated NK cells had a 2.5-fold increase in non-miRNA small RNA content (Wang et al., 2012).

**Table 1:** Selected up- or down-regulated miRNAs during cytokine activation of NK cells.

	Fehniger et al. (2010) (mouse)	Wang et al., 2012 (human)	Liu et al., 2012 (human)
Stimulation	IL-15	IFN-α	IL-2, IL-15, or IL-21
Duration (hour)	24	12 or 24	36
Up-regulated	mmu-miR-188-5p		
	mmu-miR-339-5p	hsa-let-7b	hsa-miR-15a
	mmu-miR-19a		hsa-miR-155
Down-regulated	mmu-miR-223	hsa-miR-378	
	mmu-miR-26b	hsa-miR-103	hsa-miR-1246
	mmu-miR-181a	hsa-miR-30e	hsa-miR-331-3p

*miRNAs were selected based on the following criteria: top threefold change within the top 12 expressed miRNAs (Wang et al., 2012), significant miRNAs consistently changed by the listed stimulation (Liu et al., 2012), and top threefold change (Fehniger et al., 2010).*

From the Fehniger et al.’s (2010) and Wang et al.’s (2012) data, miRNA expression patterns common to both human and mouse NK cells are evident, with miR-21 being the most highly expressed miRNA in both studies. However, there are relative abundance differences, even among highly expressed miRNAs. One potential conclusion is that miRNAs have species-specific expression patterns in NK cells. However, such cross-study comparisons of absolute abundance are difficult, since the reported human miRNA expression profiles were from peripheral blood NK cells from a single human donor, and the mouse profiling focused on splenic NK cells. Further, there may be technical differences in alignment, sequence filtering, and normalization. Contrasting mouse and human NK cell miRNA expression will require additional data from each species, ideally performed using similar sequencing-analysis pipelines.

In summary, while the basic resting NK cell miRNA transcriptomes have been reported, our knowledge remains incomplete in both mouse and human NK cells, hampered in part by a lack of extensive cross-platform validation across biological replicates. A number of questions are yet to be answered. Do different subsets (e.g., CD56^bright^ and CD56^dim^) differ in their miRNA profiles? Does maturation stage and mode of activation affect miRNA expression? Do NK cells from different cell compartments (e.g., thymic, splenic, lung, intestine, liver, etc.) express different miRNAs? Do miRNAs differ between different innate lymphoid cells (Spits and Di Santo, 2011)? Further studies are needed to understand how miRNAs are induced in NK cells, the kinetics of miRNA alterations, and the effects of perturbed miRNA expression on target genes.

## THE IMPACT OF TOTAL miRNA DEFICIENCY ON NK CELLS

Because many miRNAs are expressed by NK cells (Fehniger et al., 2010), knockout of critical miRNA processing genes, including Drosha/DGCR8 and Dicer1 (**Figure 1**) is one approach to study the effects of “global loss” of miRNAs. The first studies of miRNA in lymphocytes utilized Dicer1 knockout mice to eliminate all Dicer1-dependent miRNAs, and were focused on T cells (Muljo et al., 2005; Chong et al., 2008). These studies reported that the global knockout of miRNA led to increased IFN-γ production, and increased activation, albeit with a severe reduction in cellular count and proliferation. Similarly, studies focused on NK cells have found comparable results (**Table 2**), with a few important differences.

**Table 2:** Summary of global miRNA-deficient (Dicer1^-/- ^and DGCR8^-/-^) or over-expression (Eri1^-/-^) NK cells.

	Sullivan et al. (2012)	Bezman et al. (2010)	Thomas et al. (2012)
Cre model	hCD2-Cre	ER-Cre	Global knockout
Gene	Dicer^fl/fl^	Dicer^fl/fl^/DGCR8^fl/fl^	Eril^-/-^
Effect on miRNAs	↓	↓	↑
Survival	↓	↓	~
Proliferation	↓	↓	↓
# NK cells	↓*	↓*	↓*
IFN-γ production	↑	↓^!^	↓^!^
CD107a	↑	↓	~
Receptor repertoire alterations	None observed	↓NKG2D	Multiple, especially ↓Ly49H/D
MCMV response	↑IFN-γ production	~IFN-γ production ↓Ly49H^+^ NK cells	~IFN-γ production ↓Ly49H^+^ NK cells
*In vitro* cytotoxicity	N/A	N/A	~

* *In particular, at latest stages of NK cell maturation*.

! *In response to activating (ITAM) receptor-mediated stimulation*.

The first model to investigate the ramifications of small RNA deletion in NK cells was an inducible (estrogen receptor, ER) Cre model combined with LoxP-flanked Dicer1 and DGCR8 alleles reported by the Lanier laboratory (Bezman et al., 2010). This strategy eliminated miRNAs in all cells in the mouse following tamoxifen treatment. Focusing on the impact of this miRNA loss on NK cells revealed increased NK cell apoptosis in the periphery, combined with a selective loss of more mature CD11b^+^CD27^-^ NK cells, and a reduction in surface expression of the activating NK cell receptor NKG2D. These findings were coupled with reduced IFN-γ production and CD107a surface expression following stimulation with anti-NK1.1, anti-Ly49H, and anti-NKp46, but not stimulation with cytokines such as IL-12 and IL-18. The authors therefore concluded that miRNAs are essential regulators of NK cell development and immunoreceptor tyrosine-based activation motif (ITAM)-based stimulation of NK cells. The authors also found that miRNA-deficient Ly49H^+^ NK cells robustly proliferated in response to murine cytomegalovirus (MCMV) infection, but did not survive, and thus were not able to function in an effective NK cell MCMV response. However, these results may have confounding factors influenced by the NK-extrinsic nature of the ER-Cre model, which results in global mature miRNA loss in all cells in the organism.

A study from our laboratory utilized a more specific Cre model (restricted to lymphocytes) to investigate the results of Dicer1-dependent miRNA loss (Sullivan et al., 2012). We used a hCD2-Cre transgene (de Boer et al., 2003), which expresses Cre from the earliest stages of NK cell development in the bone marrow. Thus, in these experiments Dicer1 was removed from NK cells [marked by concurrent yellow fluorescent protein (YFP) expression (Sullivan et al., 2012)] during the earliest stages of development, and in a lymphocyte-restricted fashion. While this method was not completely NK cell specific, it removes many potential confounders by providing Dicer1-competent hematopoietic and non-hematopoietic cells. This model confirmed the reduced numbers and percentages of NK cells shown in the Bezman et al.’s (2010) study, and reduced *in vitro* survival and proliferation, indicating that NK cell development and homeostasis are critically regulated by miRNAs. However, in contrast to the Bezman et al.’s (2010) study, hCD2-Cre × Dicer1^fl/fl^ NK cells produced *more* IFN-γ and had *enhanced* degranulation (CD107a surface expression) in response to multiple activating stimuli. These effects were also apparent in Dicer1^fl/wt^ mice, showing that even decreased Dicer1 levels can have a functional consequence on NK cell biology. Further, these findings in hCD2-Cre mice were corroborated by increased IFN-γ production *in vivo* during MCMV infection. The different phenotypes in these models likely reflect different Cre-excision specificity and timing. Recently, NK cell-specific Cre models driven by the NKp46/Ncr1 promoter in a bacterial artificial chromosome (BAC) transgene (Eckelhart et al., 2011), or knock-in (Narni-Mancinelli et al., 2011) have been reported. Thus, the tools are finally available to definitively evaluate the cell-intrinsic effects of both global and specific miRNA loss- and gain-of-function in NK cells.

Another study by Thomas et al. (2012) focused on Eri1, an exoribonuclease that degrades miRNAs and thus functions as a negative regulator of miRNA-mediated control, and the effects of its loss on NK and T cells. The authors found that Eri1-deficient NK and T cells displayed an *increase* in total miRNA abundance. NK cells seemed particularly susceptible to the effects of Eri1 loss, and displayed decreased percentage and numbers, especially at the latest stages of development. The Eri1-deficient NK cells displayed an altered cell receptor repertoire, including altered Ly49H expression. In addition, while Eri1^-/-^ NK cells did not show a defect in IFN-γ production in response to IL-12 and IL-18, they produced less IFN-γ in response to ITAM-dependent activating receptors. Eri1-deficient NK cells also displayed decreased proliferation in response to MCMV infection, with increased viral titers, demonstrating the importance of Eri1 (probably due to miRNA alterations) in the context of viral infection. While Eri1-deficient NK cells have changes in global miRNA expression and a clear development, maturation and functional phenotype, one caveat to these findings acknowledged by the authors is that other RNA species are affected by Eri1, thereby providing alternative explanations for the NK cell phenotype. In any case, this study clearly implicates Eri1-mediated RNA processing in NK cell development and functional responses, probably reflective of global miRNA changes in NK cells.

Thus, the preponderance of evidence suggests that miRNAs promote cellular survival, maturation, and proliferation, while suppressing the production of key immune cytokines such as IFN-γ. However, the study by Thomas et al. (2012) suggests that miRNA-mediated repression of genes is required in both directions, i.e., increased miRNA expression can also affect NK cell homeostasis, supporting a role of miRNAs as “tuners” of cellular homeostasis. The effects of total miRNA elimination or increase on specific functions of NK cells, however, are difficult to extricate from effects on survival, and thus studying the cell-intrinsic effects of individual miRNAs in NK cells will, in the future, be a more productive approach to identifying the effects, targets, and mechanisms of specific miRNAs. One key caveat to these global miRNA alteration studies is that the models utilized are not NK cell specific and may affect progenitors and mature NK cells at different points in development/differentiation, as well as cells that interact with NK cells. Combining NK cell-specific Cre models that are now available (Eckelhart et al., 2011; Narni-Mancinelli et al., 2011) with miRNA floxed alleles will provide important confirmatory studies of how both global deficiency and specific miRNA alterations regulate NK cell biology.

## miRNA REGULATION OF NK CELL DEVELOPMENT

Like other hematopoietic cells, NK cells originate from stem cells within the bone marrow. As they develop, they require the expression of a number of signaling proteins and transcription factors essential for the NK lineage (Colucci et al., 2003; Yokoyama et al., 2004; Di Santo, 2006; Freud and Caligiuri, 2006). As discussed previously, Dicer1 deficiency in NK cells results in altered NK cell development; however, few studies have examined the role of specific miRNAs. To date, two miRNAs and their targeted molecules have been implicated in this process: miR-150 targeting c-Myb, and miR-181a/b targeting NEMO-like kinase (NLK).

miR-150 is a lymphocyte-restricted miRNA, and its expression correlates with cell maturity, suggesting a role in development (Monticelli et al., 2005). In mice, miR-150 expression acts as a fine-tuner of B cell production through the targeting of c-Myb, a transcription factor involved in hematopoiesis (Xiao et al., 2007; Zhou et al., 2007). In contrast, miR-150 over-expression only modestly reduces mature T cell numbers, in part through c-Myb and possibly Notch3, a receptor involved in thymocyte development (Ghisi et al., 2011). Bezman et al. (2011) reported that miR-150^-/-^ mice have a deficiency of mature NK cells resulting from defects in maturation and proliferation, and additionally that mice over-expressing miR-150 had an accumulation of mature, hyperfunctional NK cells. This phenotype was mimicked by c-Myb^+/-^ mice, the putative target of miR-150.

Two miR-181 family members, miR-181a and miR-181b, are also expressed in lymphocytes in a stage-specific manner. Over-expression in murine models results in increased B cell (Chen et al., 2004) and T cell numbers (Li et al., 2007). Using an *in vitro* NK cell differentiation system, Cichocki et al. (2011) demonstrated that miR-181 expression increases in parallel with the maturation stages of human NK cells. *In vitro* differentiation of CD34^+^ progenitor cells transduced by lentivirus engineered to over-express miR-181a enhanced the development of CD56^+^ NK cells. One possible mechanism for this effect is through targeting of NLK, a negative regulator of the Notch pathway, which is essential for lymphocyte development. Notch signals have been shown to support *in vitro* human NK cell differentiation (Bachanova et al., 2009; Beck et al., 2009; Haraguchi et al., 2009), and this work demonstrates a role for miR-181 family members in regulating NK cell development, possibly through modulating NLK and Notch in lymphoid or NK cell progenitors. It will be important to further assess miR-181a/b, as well as other miRNAs that change in expression during NK cell maturation, for additional mRNA targets and impact on NK cell development and maturation.

## miRNA REGULATION OF NK CELL FUNCTION

Cytotoxicity is a major function of NK cells, and multiple studies have investigated miRNA regulation of this pathway. Work on miRNAs regulating cytotoxicity has focused on the two major granule proteins, granzyme B (GzmB) and perforin (Prf1). Studies from our laboratory identified that both GzmB and Prf1 mRNA transcript was readily produced in resting murine NK cells, and was only modestly up-regulated following IL-15 activation (Fehniger et al., 2007). This was in contrast to protein levels and cytotoxic capacity, which both increased dramatically during activation, suggesting a post-transcriptional mechanism of regulation.

**FIGURE 3:**
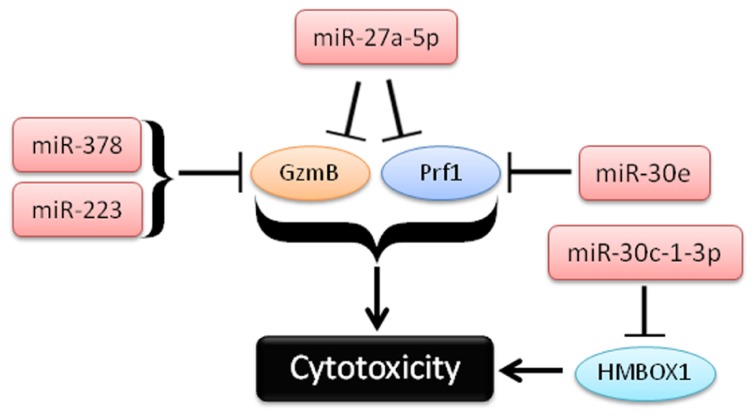
**Schema of recent studies demonstrating the role of miRNAs in repression of cytotoxicity by NK cells**.

Four recent studies have identified miRNAs regulating GzmB and Prf1 in NK cells (**Figure 3**). In studies from our lab, miR-223 was found to target the murine GzmB 3′UTR, and identified as the most down-regulated miRNA following IL-15 treatment, which we hypothesized could lead to increased GzmB production after activation (Fehniger et al., 2010). However, NK cells from miRNA-223^-/-^ mice (Johnnidis et al., 2008) appear to express similar amounts of GzmB as wild type mice at baseline, and exhibit levels of cytotoxicity similar to wild type controls, suggesting that miR-223 alone does not play a non-redundant role in limiting murine GzmB translation in resting mouse NK cells (Leong and Fehniger, unpublished data). Additional studies are still needed to determine the impact of miR-223 gain- and loss-of-function, in combination with other miRNAs, in primary NK cells.

In a second study, Kim et al. (2011) used *in vitro* differentiated human NK cells (CD34^+^ hematopoietic progenitors cultured for several weeks in NK cell differentiating conditions) to demonstrate that miR-27a-5p (miR-27a*) targeted the 3′UTRs of both GzmB and Prf1. This group observed reduced GzmB and Prf1 protein with miR-27a-5p over-expression in this system (Kim et al., 2011). Activation with IL-15 paradoxically leads to up-regulation of this miRNA, possibly serving as a negative feedback loop on GzmB and Prf1 production. Indeed, inhibition of miR-27a-5p in these differentiated NK cells resulted in enhanced *in vitro* killing and tumor protection in a xenograft model. However, the miRNA and mRNA expression of *in vitro* differentiated NK cells that result from long-term culture with high levels of activating cytokines may be significantly different than freshly isolated primary human NK cells. miR-27a-5p (miR-27a*) is expressed at very low levels in resting mouse (Fehniger et al., 2010) and human NK cells (Liu et al., 2012), suggesting a more limited role for this miRNA outside of extensive cytokine activation or *ex vivo* culture. Confirmatory studies using freshly isolated NK cells would be helpful to verify the role of miR-27a-5p outside the context of extensive *ex vivo* culture.

In the third study, Gong et al. (2012) show a role for miR-30c-1* in targeting HMBOX1 and regulating cytotoxicity. The authors use the human NK cell line (NKL), and showed that stimulation through NKG2D led to down-regulation of a number of miRNAs (Gong et al., 2012). One of these miRNAs, miR-30c-1-3p (miR-30c-1*), targeted HMBOX1, an inhibitory transcription factor of cytokine secretion. Over-expression or inhibition of miR-30c-1-3p resulted in altered killing against two hepatoma cell lines. As in the previous study, it will be important to further elucidate the role of miR-30c-1* in primary NK cells.

In the fourth study, Wang et al. (2012) demonstrate a role for miR-378 and miR-30e in repressing baseline protein expression of GzmB and Prf1, respectively. In contrast to the GzmB and Prf1 suppression model of miR-27a-5p, mature miR-378 and miR-30e expression is reduced upon IFN-α stimulation, releasing repression of GzmB and Prf1 and enhancing the cytotoxicity of NK cells. Expression of both miRNAs was shown to be inversely correlated with GzmB and Prf1 protein production. Further, nucleofection of miRNA sponges against either miRNA-378 or miR-30e in NK-92 cells led to enhanced cytotoxicity against K562 tumor targets. Collectively, these studies provide evidence that cytotoxic effector molecules, including GzmB and Prf1, can be modulated by NK cell miRNAs. Since differences in 3′UTRs exist between human and mouse GzmB and perforin, careful evaluation of each candidate miRNA in both species will be required to fully understand its role in regulating NK cell cytotoxicity.

IFN-γ is a key immune cytokine produced by NK cells following activation. The regulation of its production is complex and involves the integration of various signaling pathways (Vivier et al., 2008) and multiple layers of regulation (Young, 1996). As one form of regulation, IFN-γ is regulated post-transcriptionally via a 5′ pseudoknot and 3′ AU-rich elements, both of which can affect the stability of the IFN-γ transcript (Khabar and Young, 2007). Recently, miRNA-mediated regulation has also been reported. However, due to the integrative nature of IFN-γ production, many of these effects are indirect, instead mediating upstream signaling components. For example, signal transducer and activator of transcription 4 (STAT4), a key mediator of IL-12-induced signals, was reported to be targeted by miR-132, miR-212, and miR-200a (Huang et al., 2011). In addition, lentiviral over-expression of miR-181a/b has been reported to increase human NK cell IFN-γ production after stimulation with IL-12 and IL-18 production (Cichocki et al., 2011). However, these cells were cultured for 14 days in IL-15, transduction efficiency and subset biology was not presented, and thus the alterations may be influenced by the changes that result from long-term culture. In T cells, multiple miRNAs, including the miR-17~92 cluster (Jiang et al., 2011) and miR-21 (Lu et al., 2011b) have been shown to regulate the decision between T helper type 1 (T_h_1) and T_h_2 lineages, which has an indirect effect on IFN-γ production. Three miRNA families have been found to specifically target IFN-γ or genes immediately upstream of its induction in NK cells: miR-155, miR-29, and miR-15/16.

miR-155 was discovered to be the functional product of the BIC non-coding RNA, which is highly expressed in human lymphoma samples (Eis et al., 2005). MiR-155 has been shown to play a role in B cell (Rodriguez et al., 2007; Thai et al., 2007; Vigorito et al., 2007; Teng et al., 2008), T cell (Kohlhaas et al., 2009; O’Connell et al., 2010a), dendritic cell (Lu et al., 2011a), and macrophage (O’Connell et al., 2007) biology, particularly in the context of activation. While miR-155 is modestly expressed in resting peripheral mouse and human NK cells, its expression is markedly increased by activation with IL-18 (Fehniger et al., 2010; Trotta et al., 2012; Sullivan, Leong, and Fehniger unpublished data). Recently, Trotta et al. (2012) reported miR-155 as a positive regulator of NK IFN-γ production following IL-12 and IL-18 stimulation of human NK cells and lines that were transduced with lentivirus to over-express miR-155. This effect was reported to be mediated through direct repression of Src homology 2 domain-containing inositol-5-phosphatase 1 (SHIP-1; O’Connell et al., 2009), an established negative regulator of IFN-γ production (Trotta et al., 2012). Additional studies clarifying the *in vivo* mechanistic role of miR-155, especially in the context of *in vivo* infection and tumor models, will be of high interest. In addition, other miR-155 questions still remain unanswered: is SHIP-1 the only target of miR-155? Are there different mRNA targets before and after cytokine activation? Thus, while miR-155 has a clear role in regulating IFN-γ production, and targets SHIP-1, other roles for miR-155 in NK cell effector function remain open to investigation.

The miR-29a/b/c family is moderately expressed in resting NK cells (Fehniger et al., 2010), and has been shown to regulate the IFN-γ pathway by two independent laboratories (Ma et al., 2011; Steiner et al., 2011). However, these studies differ in their proposed mechanisms of regulation. Indeed, the two studies identify two unique targets for miR-29. Steiner et al. (2011) showed, through an extensive miRNA screening approach, that at least in CD4^+^ T cells, miR-29’s regulation of IFN-γ production was indirect, via targeting Tbx21 (T-bet) and Eomes, two important transcription factors that induce IFN-γ mRNA. In contrast, Ma et al. (2011) created an *in vivo* sponge model targeting the miR-29 family. The result of this “sponge,” which prevents miR-29 from binding to its usual targets, resulted in increased IFN-γ protein production (Ma et al., 2011). Further, through *in vitro *testing, the group showed that this regulation of IFN-γ was direct, and that T-bet and Eomes were *not* targets of miR-29. Additional *in vitro* studies by our lab in 293T cells utilizing luciferase sensor-plasmids failed to confirm the murine IFN-γ 3′UTR as a direct target of miR-29, while validating targeting of T-bet and Eomes (Sullivan et al., 2012; Sullivan and Fehniger, unpublished). Further study will be required to clarify the precise role of miR-29 in IFN-γ regulation in NK cells using NK cell-specific genetic models.

The miR-15/16 family is encoded from two distinct loci in the genome, miR-15a/16-1 and miR-15b/16-2, which are processed to three mature miRNAs: miR-15a, miR-15b, and miR-16. This family has been implicated in the development or progression of several malignancies, including chronic lymphocytic leukemia (Klein et al., 2010; Iorio and Croce, 2012). Additionally, they have been shown to regulate cell survival and cell cycle progression through regulation of Bcl2 and cyclin D1, respectively (Cimmino et al., 2005; Ofir et al., 2011). All members of the miR-15/16 family are highly expressed in NK cells, suggesting that transcription operates from both loci (Fehniger et al., 2010). These family members are predicted through bioinformatic algorithms to target the murine IFN-γ 3′UTR, which was confirmed *in vitro* by luciferase sensor-plasmid experiments (Sullivan et al., 2012). Furthermore, mature miRNAs from this family are down-regulated in primary NK cells after activation with IFN-γ stimulating conditions. While these findings suggest that the miR-15/16 family of miRNAs may be key players in regulating NK cell IFN-γ production, additional studies utilizing *in vitro* and *in vivo* loss- and gain-of-function will be required to define the non-redundant role of this miRNA family on NK cell IFN-γ production.

## NK CELL miRNAs IN DISEASE

Natural killer cells are primarily defined by their role in preventing disease and malignancy through recognition of target cells (Orange and Ballas, 2006), and a large and growing body of literature on the miRNA-mediated regulation of NK cell receptor ligands on target cells has been extensively covered elsewhere (Elias and Mandelboim, 2012). However, dysregulated NK cells can cause or participate in a wide variety of diseases, including hemophagocytic lymphohistiocytosis (Filipovich, 2009), pre-eclampsia (Riley and Yokoyama, 2008), atopy, and autoimmunity, among others. Data for the role for NK cell-intrinsic miRNA in these diseases is limited, but an important area of active investigation. For example, it was recently found that in patients with chronic fatigue syndrome, certain highly expressed NK miRNAs, such as miR-21, were significantly decreased in NK cells (Brenu et al., 2012). Most of the existing literature regarding the role of NK miRNAs in disease focuses on NK cell malignancies. Rare cancers arising from NK cells can take multiple forms with various degrees of aggression, from chronic NK cell lymphocytosis, to aggressive nasal-type NK/T cell lymphoma, to highly aggressive NK cell leukemia/lymphoma (Ishida and Kwong, 2010; Watters et al., 2011; Semenzato et al., 2012).

Several reports have shown dysregulated miRNA levels in NK cell malignancies. Yamanaka et al. (2009) reported over-expression of both miR-21 and/or miR-155 in malignant NK cell lines as well as primary patient samples. The authors further linked miR-21 to phosphatase and tensin homolog (PTEN) and miR-155 to SHIP-1, two well known tumor regulators, the suppression of which could enhance cancer progression (Yamanaka et al., 2009). In contrast, Ng et al. (2011) showed that in nasal-type NK/T lymphomas, miRNAs (miR-26a/b, miR-28-5p, miR-101, miR-146a, miR-363) were predominantly down-regulated. Whether or not this is an important biological difference between nasal-type NK/T lymphomas and the more aggressive NK cell leukemia/lymphoma remains to be established. Decreased expression of miR-146a was also found to be a poor prognostic factor in NK/T lymphoma by Paik et al. (2011). The authors suggest that this occurs via miR-146a suppression of tumor necrosis factor receptor-associated factor 6 (TRAF6), which is a target of miR-146a. miRNAs produced by Epstein–Barr virus (EBV) may be involved in the transformation leading to the aggressive NK cell lymphoma. Ramakrishnan et al. (2011) profiled NK cell lymphoma cell lines, and found that EBV-encoded miRNAs (BART-7, BART-9, BART-17-5p) were abundantly expressed, and that loss of these miRNAs resulted in reduced cell division rates. However, these intriguing results were highly dependent on the cell line chosen, and require confirmation in additional cell lines as well as primary samples. Further studies will also be required to determine the requirement, if any, for EBV-encoded miRNAs in the pathogenesis of EBV-infected NK tumor cells. In a recent study from the Caligiuri laboratory the mechanisms whereby chronic exposure to high levels of IL-15 may lead to large granule lymphocyte (LGL) leukemia were defined. Excessive IL-15 signals led to Myc-mediated suppression of miR-29b, in turn leading to Dnmt3b over-expression and DNA hypermethylation (Mishra et al., 2012). The authors showed that miR-29b inhibition directly led to increased LGL transformation in a cell line system. Furthermore, using liposomal bortezomib (a proteasome inhibitor), the authors were able to dramatically enhance the levels of miR-29b, which was correlated with decreased Dnmt3b expression and disease-free survival in ICR-SCID mice that were normally 100% susceptible to LGL leukemia. These studies directly implicate loss of miR-29b in causing LGL leukemia with rescue of miR-29b levels resulting in resolution of disease. Thus, this provides the rationale for studying miR-29 mimetics as a novel miRNA-based therapy for this disease.

There remain many unanswered questions in how miRNA dysregulation may lead to disease. For each of the miRNAs putatively involved in NK cell disease pathogenesis, it will be important to study the role of these miRNAs in primary cells and model organisms using both genetic gain and loss of miRNA function in order to address the mechanism of miRNA function. As of now, much of the data for miRNA involvement in disease is correlative, but if miRNA alterations are shown to cause or contribute to pathogenesis or disease progression, there exists great potential for targeting using miRNA mimics or silencers *in vivo*.

## miRNA TARGET DETERMINATION IN NK CELLS

miRNA biogenesis is regulated at multiple levels, and private components have been identified that may control individual miRNA expression levels (Kim, 2005; Winter et al., 2009). Further, many miRNAs exist in families, with closely related members that share key binding specificity sequences, including the “seed” sequence, from base pairs 2–8 (5′ → 3′) of the mature miRNA, that is the major determinant of target specificity (Garcia et al., 2011). These families of miRNAs are thought to cooperate in their regulation of target genes, and thus loss-of-function models can be difficult, as one must account for all expressed family members of a given family. In order to avoid this complexity, miRNA “sponges” that sequester whole miRNA families can be transgenically introduced; however, it is possible that introducing a foreign small RNA sequence could have unintended or off-target consequences.

miRNAs can be computationally predicted to bind to hundreds of target mRNAs (Lewis et al., 2003), but these predictions are prone to false positive and negatives, and thus it is critical to biochemically and biologically validate predicted miRNA:mRNA target interactions. In addition, multiple disparate mature miRNAs likely regulate the same mRNA via distinct and/or overlapping 3′UTR binding sites, and it remains unclear how these multiple sites interact. A plethora of miRNA target determination tools have become available over the past few years, including bioinformatic algorithms such as TargetScan^1^, Ensembl MicroCosm^2^, and others (Wang, 2008). These tools are useful in developing initial hypotheses about the role of individual miRNAs in cellular function, but have a high percentage of false positives (Neilson et al., 2007; Sullivan, Leong, and Fehniger, unpublished data); i.e., many “targets” determined by bioinformatic algorithms may not be true targets *in vitro* or *in vivo*. An ostensibly straightforward molecular biology experiment to determine if a gene is targeted by a miRNA is to transduce a target cell type with the miRNA of interest, and observe a down-regulation at a protein level via western blot or equivalent method. However, this approach is complicated in NK cells by the difficulty in reproducibly introducing foreign RNA into resting NK cells, as well as off-target effects of miRNA that may result in the observed repression being an indirect, rather than direct effect. Therefore, though prediction methods and *in vitro* transduction can be useful for hypothesis generation, they are surprisingly limited in their ability to validate *in vivo* miRNA:mRNA interactions.

**FIGURE 4:**
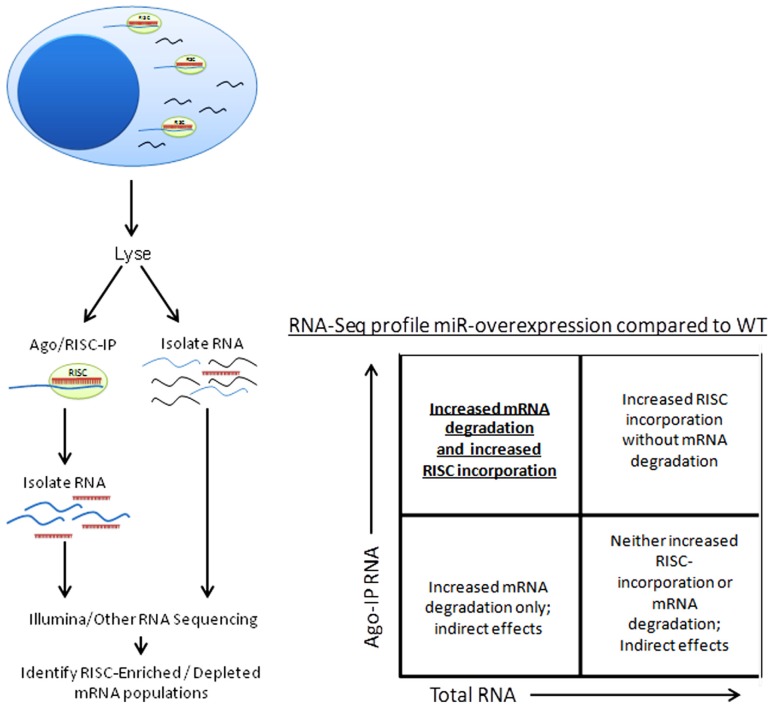
**Schema of Argonaute/RISC immunoprecipitation procedure and analysis**.

Besides the obvious benefit of phenotype confirmation, creation of a miRNA-knockout or transgenic mouse model grants the opportunity to use a variety of techniques to confirm individual miRNA targeting of specific genes. Argonaute immunoprecipitation is one effective method of screening for the effects of an individual miRNA on a specific cell type (Karginov et al., 2007; Chi et al., 2009; Matkovich et al., 2011). In this method, both total RNA and RNA immunoprecipitated using anti-Argonaute (a component of the RISC) antibodies, and are sequenced (using next-generation sequencing approaches) and aligned to the genome (**Figure 4**). miRNA-knockout or miRNA-over-expressing NK cells can be compared to WT NK cells for their RISC-bound (**Figure 1**) RNA “profile,” as well as their total RNA profile. While controversy still exists in the field over the relative contribution of miRNA to transcript degradation or translational inhibition (Ambros, 2004; He and Hannon, 2004; Liu, 2008; Selbach et al., 2008), a combination of assessing total RNA changes and RISC-incorporation changes can determine, at a functional level, the role of an individual miRNA in targeting. This technique has yet to be applied to NK cells, and will likely unveil very interesting findings.

Predicted miRNA targeting of an mRNA can also be biochemically validated, most commonly using a luciferase “sensor plasmid” reporter system. In this system, the 3′UTR of a putative target gene is fused to luciferase. This construct is then transfected into a cell line, such as HEK293T, while co-transfecting another vector that over-expresses or inhibits the miRNA of interest. If a miRNA targets the 3′UTR of interest, this is experimentally measured by a decrease in luciferase activity. This approach, while useful, has a number of caveats, and it is important to be cognizant of potential confounders in this system. Proper controls and verifications must be included, including (1) validation of miRNA over-expression, (2) use of a luciferase vector with no 3′UTR to determine “baseline” targeting of the vector, (3) normalization of the luciferase signal to control for transfection efficiency differences between different luciferase constructs, and (4) mutation of the putative miRNA binding site in the 3′UTR in order to demonstrate that loss of this predicted site results in loss of miRNA-mediated repression. The last issue, mutating the predicted binding site, is critical to demonstrate a *direct* targeting event. In our experiments, the psiCheck2 vector (Promega) has performed consistently and effectively. The use of a non-NK cell to evaluate a mRNA:miRNA targeting interaction is a reasonable first step in validating that the miRNA targets the given mRNA. In fact, since some miRNAs that are highly expressed in NK cells are not highly expressed in 293T cells, these miRNAs may be more easily over-expressed and otherwise manipulated, allowing one to more easily observe the effects of miRNA over-expression on that specific target. However, similar to most functional aspects of NK cell biology, it is critical to validate a given miRNA:mRNA targeting event in primary NK cells, since there is the possibility that certain effects of miRNAs require inducible machinery that is unique to NK cells. This requires specific miRNA mouse genetic models or *in vitro* mediated gain- and loss-of-function, paired with evaluation of the specific miRNA-mediated phenotype in primary NK cells.

## CONCLUSION

miRNAs are a class of non-coding RNAs that have increasingly been shown to be critical to NK gene regulation. Recently, the NK cell field has become acquainted with the miRNA expression profile of murine and human NK cells, the global role of miRNAs in NK cell biology, and the influence of a few individual miRNAs on the NK cell molecular program in homeostasis as well as disease states. However, with over 60 highly expressed miRNAs in NK cells, there is still much work to do to dissect the function of each individual miRNA in these unique immune cells.

## Conflict of Interest Statement

The authors declare that the research was conducted in the absence of any commercial or financial relationships that could be construed as a potential conflict of interest.

## Footnotes

1 http://www.targetscan.org/

2 http://www.ebi.ac.uk/enright-srv/microcosm/htdocs/targets/v5/
